# F-DCS: FMI-Based Distributed CPS Simulation Framework with a Redundancy Reduction Algorithm

**DOI:** 10.3390/s20010252

**Published:** 2020-01-01

**Authors:** Seokjoon Hong, Ducsun Lim, Inwhee Joe, WonTae Kim

**Affiliations:** 1 The Department of Computer and Software, Hanyang University, 222 Wangsimni-ro, Seoul 04763, Korea; daniel379@hanyang.ac.kr (S.H.); imcoms@hanyang.ac.kr (D.L.); iwjoe@hanyang.ac.kr (I.J.); 2 The Department of Computer Science and Engineering, Korea University of Technology and Education, Cheonan-si 31253, Korea

**Keywords:** cyber-physical system, electric vehicle, distributed co-simulation, functional mock-up interface, zero crossing, driving cycle

## Abstract

A cyber physical system (CPS) is a distributed control system in which the cyber part and physical part are tightly interconnected. A representative CPS is an electric vehicle (EV) composed of a complex system and information and communication technology (ICT), preliminary verified through simulations for performance prediction and a quantitative analysis is essential because an EV comprises a complex CPS. This paper proposes an FMI-based distributed CPS simulation framework (F-DCS) adopting a redundancy reduction algorithm (RRA) for the validation of EV simulation. Furthermore, the proposed algorithm was enhanced to ensure an efficient simulation time and accuracy by predicting and reducing repetition patterns involved during the simulation progress through advances in the distributed CPS simulation. The proposed RRA improves the simulation speed and efficiency by avoiding the repeated portions of a given driving cycle while still maintaining accuracy. To evaluate the performance of the proposed F-DCS, an EV model was simulated by adopting the RRA. The results confirm that the F-DCS with RRA efficiently reduced the simulation time (over 30%) while maintaining a conventional accuracy. Furthermore, the proposed F-DCS was applied to the RRA, which provided results reflecting real-time sensor information.

## 1. Introduction

Along with the progress and innovations in information and communication technology (ICT) in recent years, its application range has been gradually expanding. A cyber physical system (CPS) is a distributed control system in which the cyber part and physical part are tightly interconnected and it is defined as a next-generation paradigm that has real-time and intelligent characteristics as well as predictability and advanced adaptability [1,2,3,4]. A CPS can realize seemingly impossible tasks by tightly combining physical objects, including machines, automobiles, factory facilities, and humans, with technologies defined as the cyber part. It can acquire various types of information through monitoring by using sensors in heterogeneous devices and facilitate interaction and collaboration, by which information is shared with other sensors and actuators through a network. Based on such shared information, it facilitates accurate analysis for the physical part and appropriate operation of desired functions in order to use them efficiently. Accordingly, cyber-physical systems (CPS) are being applied to a broad range of research areas, such as intelligent transport systems, smart grids, medical equipment, smart manufacturing systems, nuclear power plants, aviation, and the automobile industry [5,6,7,8,9]. In the automobile industry, a CPS is usually applied as a system that monitors and controls a combination of sensors, actuators, and physical components through a network. In the rapidly growing present society, academics and industries are adopting plans of using green energy, such as hydrogen, as solutions to reduce environmental problems, transportation problems, and dependency on conventional fossil fuels [10,11].

In this regard, cyber physical energy systems (CPESs), an extension of CPSs, are applied to the energy domain [12,13]. A CPES consists of an energy resource, network, application, and consumers, and based thereupon, the requirements for next-generation energy are satisfied. The goal of CPES is to further enhance real-time sensing and dynamic monitoring in a distributed system and wide area network (WAN) environment with respect to the use of an energy resource [14]. New CPES-related information technologies have been applied to electric vehicles (EVs), smart grids, home automation systems, etc., with the aim of achieving efficient energy usage and environmental protection. Tehrani and Maurice [13] presented a design for a cyber energy system to enhance the autonomy of EVs. Kim and Mosse [15] provided a general framework for the design, modeling, and simulation of a CPS.

A conventional vehicle system consists of mechanical elements such as an engine and transmission. In an electric vehicle (EV), however, ICT is applied in the form of software and communication technologies, which are combined with electrical parts such as battery management systems (BMS) and electric machine; they are monitored and controlled through feedback loops of sensing and actuation. Therefore, the best approach towards improving the efficiency performance prediction of EVs would be to optimize the system and reduce the development time through CPS modeling based on combined ICTs. This can be accomplished by selecting each component of EVs based on CPS characteristics and by collecting and applying the information. Preliminary verification through simulations for performance prediction and quantitative analysis are essential before application to a real model because a complex system comprises various highly coupled components [16,17,18,19]. Such simulations are considered very important because they can continually improve the model throughout the development process of the system model to facilitate the efficient operation of the system.

Co-simulation [20] is a simulation technique that facilitates the execution of global simulation by combining an existing simulation tool and a simulator generated from different simulation tools in a whole system. Instead of using only one type of tool and language when performing a simulation, a specialized tool called a solver (a software component that includes event processing and integration algorithm to solve a given problem in a model) is used to perform the modeling separately for independent parts of the system; then, these models are linked via integration and modification, and co-simulation is performed. In comparison with various conventional monolithic simulations, co-simulation can facilitate the independent modeling of subsystems and then integrate and modify the whole system. Therefore, co-simulation is more effective in the aspect of merging domains and reusing and sharing the model. Furthermore, co-simulation offers advantages for the verification of CPS and multi-domain systems as it provides a method of considering multiple domains with mutually different time steps.

Research on co-simulation has been conducted in various areas such as automobiles [21,22,23,24], electric power systems [25], and aviation [26]. When simulating a complex CPS model in the EV and various other areas, a user can perform the simulation by additionally using a model developed with a different simulation tool.

**Figure 1:**
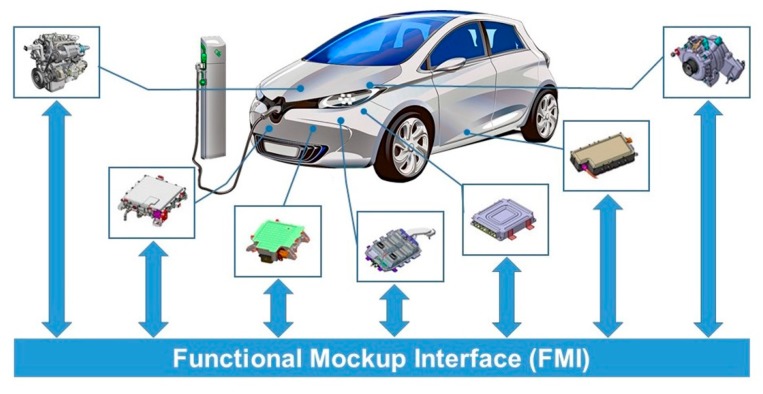
FMI-based simulation in the electric vehicle (EV) domain.

The functional mock-up interface (FMI) standard [27], which is an international standard for methods of interfaces in simulation models, supports co-simulation to facilitate easy integration of models developed through various simulation tools. The FMI defines a standardized application programming interface (API) such that simulators can operate together and a model that implements it is defined as a functional mock-up unit (FMU). The FMU is a “black box” that is equipped with input port, output port, and status variables set, and uses an API to implement the initialization of status variables, setting of the input port value and searching for the output port value. Continuously developing and managing this standard has many potential benefits. First, in general, the details included in the “black box” are not available to users. This can significantly reduce the time required for gaining knowledge on operation details and the amount of relevant knowledge required. It is thus a potentially excellent method for preserving the intellectual property (IP) of manufacturers. Therefore, a dynamic model developed in a different simulation tool needs not be converted into a model directly suitable for the host simulation tool. Secondly, it features simulator independence with respect to compiling, linking, and distributing FMU. Thus, application is possible even without the information of the target simulator. Thirdly, there is a clear possibility that a technology exclusive to a certain area can be widely applied to other areas as well by properly using the advantages of the FMI standard. In the Figure 1, the respective components were developed through EV research [28], and we implemented as virtual prototypes.

The FMI [27] standard specifies an API for interconnected FMUs and defines a master algorithm (MA) in terms of calling the API and orchestrating the co-simulation, but it does not designate any specific MA. The design and use of an appropriate MA are important because the requirements of simulation differ depending on the situation. If a simulation is performed without a necessary MA, functions for basic modeling, including simple discrete-event simulation and variable step-size numerical integration algorithm, cannot be used. The MA controls data exchange and synchronization between FMUs. The most important requirements for the implementation of the MA are accuracy and efficiency. Usually, these two requirements are contradictory. In a simulation, the MA can reduce the allowable error by improving the accuracy through repeated steps, which significantly increases the runtime. If a step is not repeatedly performed, the efficiency can be improved by increasing the step size according to the step performed. This will, however, decrease the accuracy. Therefore, despite its important role, it is difficult to devise an MA.

Accordingly, attempts have been made to design MAs having the characteristics of accuracy and efficiency [29,30]. In Lee [29], step determination was considered a major characteristic of the MA. In their simulation, the largest step size that can be accommodated before performing the step was determined for every FMU, and among them, the minimum value was selected to determine the step size. However, even after finishing the simulation step, the step size was still found to be large. On the other hand, [30] presented a step revision that enhances the step determination, and it included rolling back to a previous timepoint before moving the step forward in the whole simulation. Based on these studies, this paper proposes an FMI-based distributed CPS simulation framework (F-DCS) adopting a redundancy reduction algorithm (RRA) for the validation of EV simulation.

The remainder of this paper is organized as follows. Section 2 describes the FMI, MA that performs the rollback and data distribution service (DDS) framework. Section 3 explains the Distributed CPS simulation framework with RRA in detail. Section 4 presents a Distributed CPS model based on hybrid modeling and Section 5 describes the validation of EV simulation of the proposed FMI-based Distributed CPS simulation framework, and lastly, Section 6 concludes the paper.

## 2. Related Work

### 2.1. Function Mock-Up Interface

The FMI standard is an independent interface tool standard developed through MODELISAR project research managed by the Modelica Association. It supports standardized data exchange between dynamic models designed in mutually different simulation environments by using C codes and xml files [27]. The main goal of FMI is to make improvements such that models designed with different modeling tools can exchange data and components or sub-systems that can interoperate. The models are packaged in the form of a black box called the FMU, which can be connected to a larger upper-level model owing to the standard interface that can access the state and derivatives included in the equations. In the abstract aspect, the FMU can be viewed as a timed Mealy machine, a finite-state machine in which the output value is determined by the status of the input value only, and an access from an outside part can interact with the FMU through the API only. The FMI standard has adopted an API that the FMU should comply with.

We introduce some essential functions of FMI that allow communications between a master and a slave when running a simulation.

- fmiDoStep: This function is provided by the FMI for a method of advancing time in co-simulation. There are three arguments in a call: currentCommunicaiton, which shows the current click time of the master; communicationStepSize, the step size that the solver has to calculate; and noSetFMUStatePriorToCurrentPoint, which shows whether the master will call/not call fmiSetFMUState for the previous communication point. If the fmiOK call is returned, the step has been successfully executed, and if it cannot be executed, fmiError is returned. If the slave returns FMIDiscard, only some parts of the communication step have been calculated, and here, the MA should be re-executed with a smaller communication step size.
- fmiSetXXX and fmiGetXXX: The MA provides input data to the FMU by calling a procedure called fmiSetXXX provided by the FMU. It calls fmiGetXXX to retrieve an output value from the FMU and provides an argument for the ID of the output and a pointer to a storage location (“XXX” is defined with the data type of the input and output).
- fmiSetFMUState and fmiGetFMUState: These functions allow the master to store and restore the whole state of the slave. fmi2SetFMUstate restores the internal state of the FMU. This requires the input pointer previously copied for the FMU state and the current state of the FMU is replaced with the copied one. fmi2GetFMUstate stores the internal state of the FMU. It receives the pointer related to the FMU state as an input and copies the current FMU state.

These functions allow the master to roll back the communication step. The implementation of these functions is optional. They deal with a legacy system. The FMU can wrap the legacy simulator that does not allow storing and restoring.

As the FMI includes various APIs which allow reading and writing of the FMU’s state, large support can be provided to the MA, but several factors need to be reinforced. First, because overhead can occur if a state is stored and restored, the design should consider a method for reducing the overhead. Secondly, because the implementation of the rollback procedure by the FMU is optional, it may be quite difficult to implement it according to the characteristics of each model. Thirdly, as shown in our research, although the APIs supported in the FMI are quite helpful, the supported APIs are not sufficient in terms of designing to prevent the occurrence of rollback in a section where it should not occur. Therefore, this study implemented the rollback function by complying with the API supported in the FMI standard, such that it will be supported in the FMU. Determining the sequence of calling these procedures is an essential factor in the design of the MA.

### 2.2. Master Algorithm for Rollback

**Figure 2:**
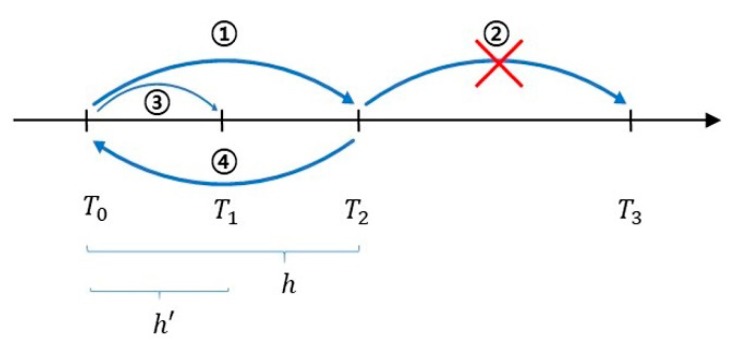
Step revision algorithm with rollback.

Many MAs for orchestrating FMI-based co-simulation have been proposed. In [29], the simulation was performed through doStep with a basic step size first for several FMUs. If an FMU has not found the ZCP, a rollback to a previous time instance is performed. Afterwards, the maximum step size that can be proceeded with by each FMU is checked, and among them, the minimum value is selected to proceed with the simulation. In contrast, [30] proposes the following method. The procedure of step revision algorithm is illustrated by the sequence in Figure 2. When co-simulation is performed using several FMUs, the simulation is carried out with the basic step size (h) first ($T_{2}=T_{0}+h$). Among them, if an invalid state or error flag occurs in any of the FMUs, the whole simulation is rolled back to the previous simulation time $T_{0}$. Then, after adjusting the step size of all the FMUs to a smaller step size (h’), the simulation is repeated until the ZCP is reached.

### 2.3. Middleware for Distributed Co-Simulation

The concept of distributed co-simulation is based on the idea that simulations are performed on multiple computers and can be distributed not only logically but also physically through a network. If distributed co-simulation is performed using more than one remote simulator, the simulation execution time can be reduced by balancing the workloads at multiple nodes through parallel tasks. Subsequently, the results of executing at distributed domains should be integrated with the framework. A middleware is used to perform the co-simulation, in which a multi-agent architecture facilitates the execution of parallel and distributed tasks.

Many open-source tools have been developed and researched for FMU simulations. Among them are tools that support distributed co-simulation of FMU, such as DACCOSIM [31] and FMI Go! [32]. DACCOSIM is an FMI-based co-simulation tool implemented in Java. A user can design and execute multi-simulation based on cooperation between multiple FMUs in multi-core or cluster environments. The technology used for exchanging data between FMUWrappers varies depending on the corresponding location. In FMUWrapper, a shared queue is used for communication to a local master, and messages are sent through the ZeroMQ middleware for communication between a local master and a global master. FMI Go! is an open-source software with permissive license, and it was designed for using components compatible with the FMI standard in the distributed simulation environment and platform. FMI-Go! uses a server-client architecture in which servers host FMU individually, and uses the ZeroMQ middleware to send messages for mapping of various FMI functions.

This paper suggests an alternative or replacement of conventional solutions from the middleware perspective, verifying the distributed co-simulation environment by using DACCOSIM, FMIGO!. We applied the data distribution service (DDS), a data communication middleware, to facilitate efficient and autonomous data exchange in various platforms and communication environments demanded by users. The DDS is a publish/subscribe-based network communication middleware and an Object Management Group (OMG) standard that supports real-time, scalable, and dependable data exchanges [33].

An application used for communication in DDS is DomainParticipant. Multiple domains can exist in a same network, but DomainParticipant can subscribe to only one of them and can be a publisher, subscriber, or both. A publisher includes one or more DataWriter, and a subscriber manages one or more DataReader. DataWriter and DataReader are bound with a topic when generated from the publisher and the subscriber, respectively. If bound with the same topic, they are interconnected logically. However, the subscriber does not need to know the location of the publisher. Multiple publishers can post for the same topic and multiple subscribers can subscribe to the same topic.

The pub/sub implemented in the DDS is a data distribution technology in which a user participating in a domain becomes a publisher/subscriber to produce and consume the desired data only, unlike the server-client system. If a large number of devices provide data frequently in a distributed manner in the distributed co-simulation, it may not be efficient. As the pub/sub distribution technology facilitates the free participation and withdrawal of domain and provides functions for producing and collecting desired data only, it can provide a suitable environment. Furthermore, because it facilitates access to data regardless of location, time, and synchronization with respect to the service, it provides a suitable environment for real-time communication. This will increase the possibility of making progress when activating connections and integration of the Internet of Things (IoT) for a digital twin.

## 3. Distributed CPS Simulation Framework with RRA

### 3.1. Distributed CPS Simulation Framework

We propose a framework consisting of DDS middleware and FMUWrapper for the FMI-based distributed co-simulation. Here, FMUWrapper is an FMI interface for simulating the FMU at each distributed node. Moreover, the DDS middleware not only sends the simulation data produced between FMUs, but also sends control messages for simulation status information and synchronization of rollback performed between one Global Master node and several Local Master nodes.

**Figure 3:**
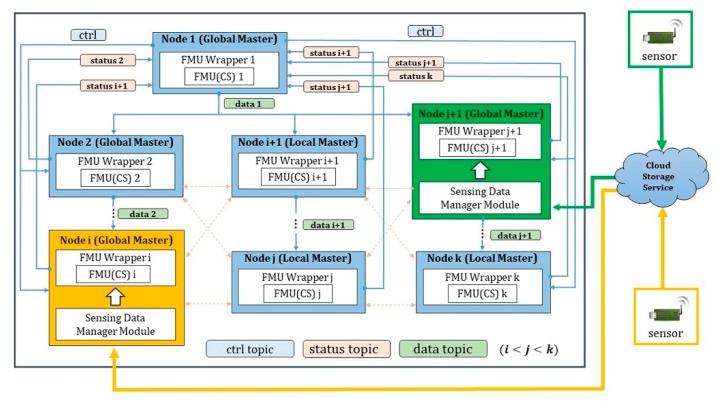
FMI-based distributed cyber physical system (CPS)-simulation framework.

In the distributed CPS simulation framework proposed in Figure 3, the distributed nodes are the Domain Participants. Moreover, a structure of Ctrl, Status, and Data types is used to transmit the control data and simulation data between the nodes. First, the Ctrl type can be used when the global node controls other local nodes. For example, the global node can use Ctrl type messages to request the starting, stopping, and rolling back of simulation to the local nodes. The Status type allows each local node to share its simulation performing status information to the global node. The Data type is used when the simulation data are transmitted between the nodes.

First, if the simulation is performed normally, the global node performs the simulation using the initial step size value for the simulation time t through FMU Wrapper and sends the result to the connected nodes through Data-type messages. A node that has received the data sets the reference value according to the data I/O connection setting between the FMUs of co-simulation; afterwards, it sets the input value with the setReal function. Here, a data message sent from the global node contains the current step size information as well and when each local node receives this message, it changes the current simulation step size with the step size from the data message and performs the simulation. Hence, the global node and local nodes are synchronized. If the simulation for each FMU is performed without any problem at the current simulation time (*t*) of all the connected nodes, the global node increases the next simulation time (*t + hh*) and performs the simulation again for the FMUs.

If a rollback occurs while performing the simulation from time t to t+hh at a certain node, the rollback to time t is performed first. Subsequently, the global node is informed about the rollback through a Status message. Then, the global node performs the rollback and sends a Rollback Ctrl message to all connected local nodes such that all nodes can be synchronized by rolling back. Among nodes that have received the Ctrl message, there may be nodes where the simulation time has not proceeded to t+hh yet. In this case, the rollback is not performed.

Furthermore, some nodes have a sensing data management module and a sensor provides the collected data from outside to the node through a cloud in real-time. FM Wrapper inputs the provided sensing information in the FMU with the setReal function, and the simulation is performed by applying the provided value. In this manner, the simulation can reflect real-time sensing information, such as temperature and humidity.

Next, a method of predicting and applying the step size at each node is examined. At each node, the step size can be determined by using the two proposed algorithms for each FMU. It is important to determine the step size that can be commonly applied to every FMU in the co-simulation, in which FMUs in multiple nodes interoperate. For this purpose, the next_hh value is used in DDS Data messages and Status messages. At the current simulation time t, the step size determined using the algorithm at each node, is stored as the next_hh value and delivered along with the simulation data in a DDS Data message from the global node to immediately before the final local node. The receiving node compares its determined step size with the next_hh value and continually updates it with the smaller value. Then, the value is delivered to the final node. When the current location is the final node, its determined step size is compared with the next_hh value and it is updated with the smaller value. Next, this value is sent to the global node through a Status message. Finally, the global node determines the step size with the minimum value among the next_hh values received from the final nodes, and the simulation of *t + next_hh* is performed.

### 3.2. Redundancy Reduction Algorithm

**Figure 4:**
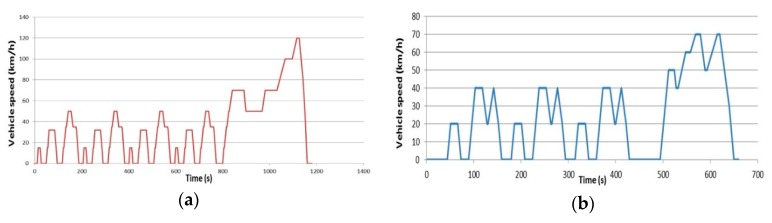
Driving cycles for EV simulation; (**a**) New European Driving Cycle (NEDC), (**b**) 10–15 mode cycle.

Among the most widely used techniques for testing combinations of vehicle components, a typical method applies a driving cycle to the simulation [34,35]. By applying a driving cycle to the simulation of an EV model, it is possible to determine the vehicle’s driving distance by time and battery SOC (state of charge) by power consumption based on the EV’s operation [36,37,38]. When several driving cycles are examined, a repetition section can be observed in a cycle, as shown in Figure 4.

**Figure 5:**
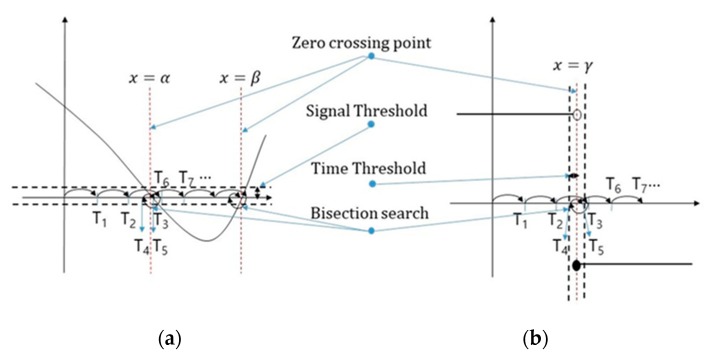
Zero crossing point and bi-section search; (**a**) continuous model, (**b**) discontinuous model.

If the model is simulated by accepting such a driving cycle as an input value and combining it with other components in the EV, the simulation graph can have positive and negative values depending on the time, and many zero-crossing points (ZCPs) [39] are produced. To find a section that has a value of 0 exactly, a bisection algorithm [40] can be used. When a ZCP occurs in the simulation of a certain model, as shown in Figure 5, this algorithm checks the associated error. If the error is larger than the signal threshold, the previous step is repeated with the step size reduced to half, as shown in Figure 5a. Here, if the simulation data value is smaller than the signal threshold, the simulation of the next step is performed; if not, the above process is repeated. Suppose the simulation is for Figure 5b, in which the graph of the simulation model does not go through the point 0 directly and the value changes from positive to negative or vice versa. Then, the time threshold can be assigned for the simulation interval ($T_{n+1}-T_{n}$) and the exact time of changing the sign can be determined accurately by using the bisection algorithm. On the other hand, if the bisection algorithm is used to find the ZCP, this method will be considerably inefficient as the simulation time will increase in the simulation model with a repeating pattern.

Considering these issues, this paper proposes a method that can reduce the simulation time more efficiently while maintaining the conventional accuracy by using the repetitive pattern information of driving cycles in an EV simulation. First, when performing a simulation, information for the simulation values is obtained through the MA and the repetition section in the driving cycle is predicted. Then, the step size is predicted using the obtained information and the simulation is performed.

This paper proposes two algorithms for reducing the time of FMU-based EV simulation using a repetitive and cyclic driving cycle.

First, for a case with a pattern from a cycle in a simulation graph, an adaptive step size algorithm is proposed to reduce the simulation time using that pattern. If the simulation is performed multiple times with a small step size as the simulation data value is repeated, it may be more efficient to perform the simulation with a large step size instead of the small step size.

Secondly, for a case with ZCP occurring in a different simulation model in the first starting cycle of the driving cycle, rollback pattern information is saved. Then, if the ZCP is predicted to occur in the same graph cycle in the model, the simulation value in the saved rollback pattern information is compared with the step size value, thereby finding a mapping step size for the current simulation value. By applying it, the second proposed algorithm can minimize the frequency of rollbacks in the simulation.

**Figure 6:**
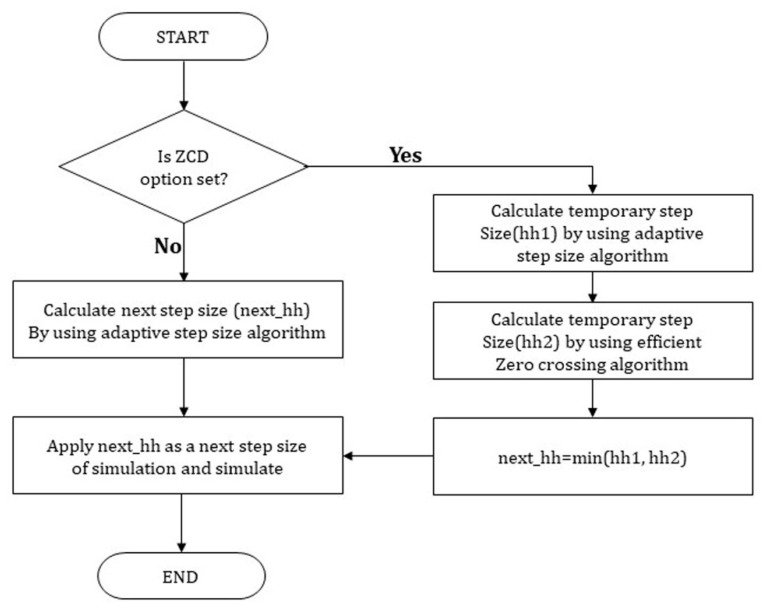
Sequence diagram of the redundancy reduction algorithm.

The sequence diagram describing this method is shown in Figure 6.

#### 3.2.1. Adaptive Step Size Algorithm

**Figure 7:**
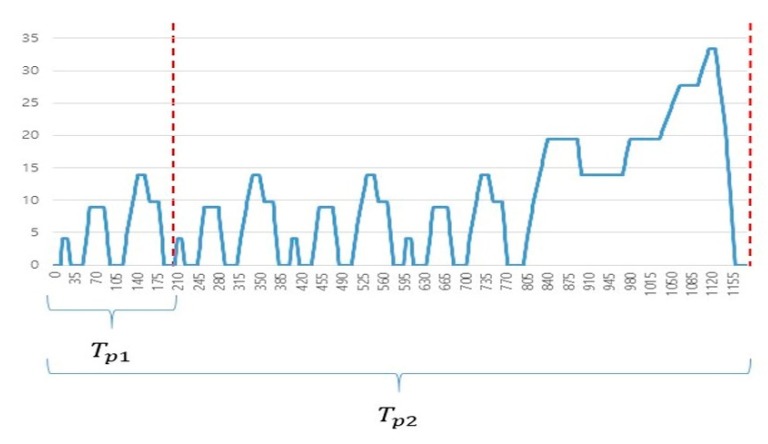
New European Driving Cycle (typical repetition cycle).

Algorithm 1 shows the process of saving level patterns in the adaptive step size algorithm. First, when the simulation begins, the step size is initialized as 1 and it checks changes by the time of simulation to be measured during the first small repetitive cycle $T_{p1}$ while performing the simulation Figure 7. Here, the step size was set to 1 initially because the data information of the driving cycle was given in 1 s intervals. If the same simulation value is repeated in every step, the length of the repeating section is checked when the repeating section is completed, and the value is stored in a structure designed to store simulation pattern information. The structure for storing pattern information has value, duration, and order information, representing the simulation value, maintained duration and the order of appearing in the cycle, respectively. Here, the duration can be obtained by subtracting the first simulation time from the last simulation time. Furthermore, when the same value appears again during the repetition cycle $T_{p1}$, the order is used to distinguish it. In other words, for a simulation value appearing first when saving in the array, the order is 0, and if the same value appears repeatedly, their orders are set to 1, 2, …, n, and stored.
**Algorithm 1:** adaptive step size algorithm (saving level patterns)
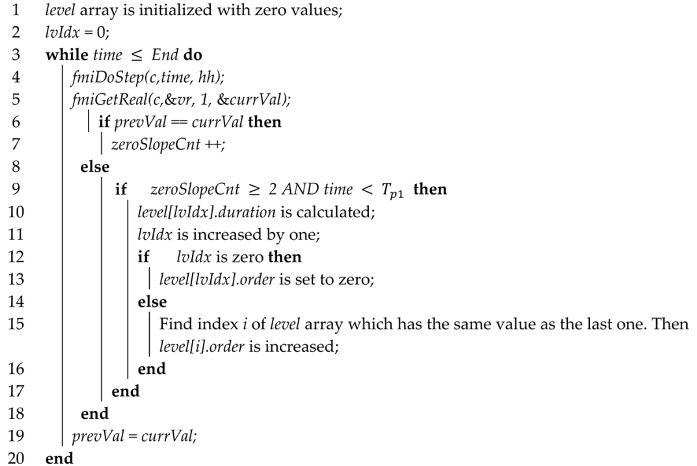


Algorithm 2 shows the process of the search for level patterns and step size application in the adaptive step size algorithm.
**Algorithm 2:** adaptive step size algorithm (search of level patterns and step size application)
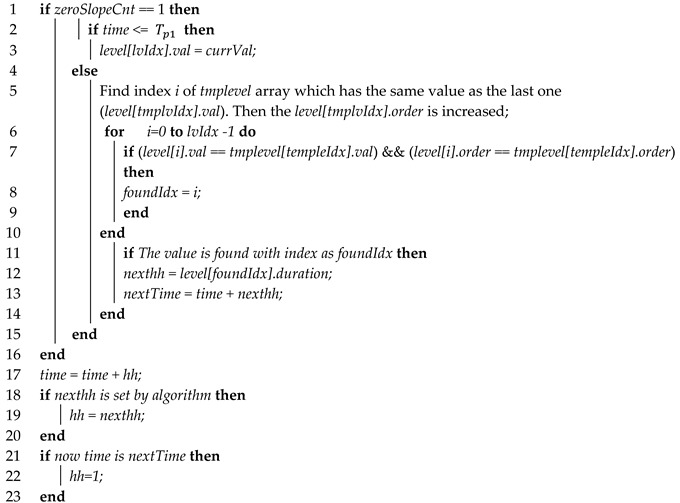


Next, if the first repetition cycle $T_{p1}$ is completed, the current value is added to the simulation pattern information using a new structure variable for pattern information comparison. If the current order is the same as the previous order, the step size is as large as the duration in the previous pattern structure information is applied. In this manner, repetitive simulation sections can be reduced. If the value is not the same, the step is set to 1 by rolling back to the previous step again, and then the simulation is performed. Usually, because the simulation values are repeated several times in $T_{p2}$ inside the driving cycle, there is a high probability that the same value will appear even after passing the step by as much as the duration. Furthermore, because the same pattern of the simulation value can reappear in the repetition cycle, the existence of the same simulation value in the stored structure is checked. If such a value exists, it is updated by adding 1 to the order in the structure. Thereafter, the existence of the same value in the previously saved pattern information and the order is checked. If there is any such value, the duration is applied as a new step size. In this manner, efficiency and speed of the simulation can be improved because the simulation can be performed by maximally expanding the step size for the same simulation value in the remaining cycles after excluding the first cycle. Furthermore, because the EV simulation method usually measures the EV driving distance or battery SOC by repeating the whole cycle $T_{p2}$, the pattern information saved during $T_{p1}$ can be continually used.

#### 3.2.2. Efficient Zero Crossing Detection Algorithm

**Figure 8:**
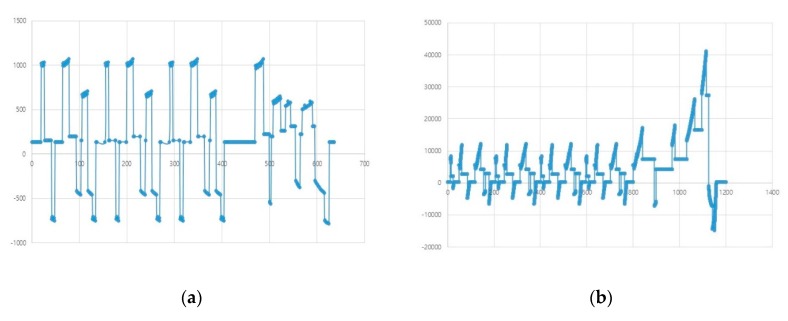
Examples of simulation using the New European Driving Cycle; (**a**) tractive effort or force F_t_ (N), (**b**) power consumption from the battery P_bc_ (W).

The second algorithm can be used in the co-simulation using ZCD and repeating rollbacks can be minimized. The ZCD technique is useful because zero crossing (ZC) can be determined. When the bi-section algorithm is used for ZCD, rollback is performed by reducing the step size by half if ZCP occurs and the error with 0 is large; rollback is stopped only if the result is below the threshold value. However, in the case of many ZCPs occurring in a short time, as shown in Figure 8, the simulation time will be lengthy due to frequent rollbacks if the bi-section algorithm is used.

**Figure 9:**
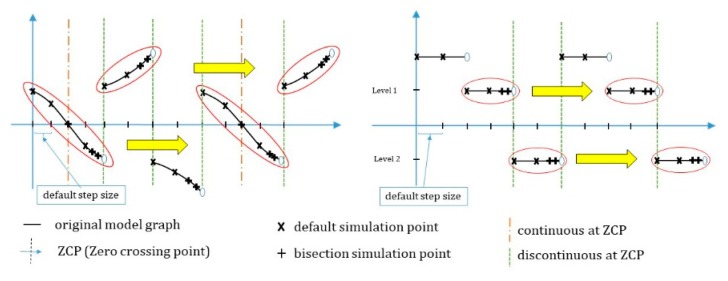
Example of zero crossing.

Therefore, the proposed algorithm saves the rollback pattern if ZCP occurs in the first repetition cycle $T_{p1}$. If ZCP occurs afterwards, a small step size is applied by using the saved rollback pattern information in order to reduce unnecessary rollbacks. To accomplish this, the proposed algorithm uses the following method. As shown in Figure 9, the cases of ZCP occurrence can be divided into two cases: occurrence in a graph of increasing or decreasing shape, and continuously maintaining the same value and then suddenly changing from a positive value to a negative value or vice versa in a stepwise manner.

First, in the case of a sequential graph shape with a slope, the simulation graph is continuous in each section; however, the same value is not maintained and the value continuously changes, as shown in Figure 7. Here, sections are divided into down crossing sections, for each of which the value is changed from a positive value to a negative value, and up crossing sections, in which a negative value is changed into a positive value.

**Figure 10:**
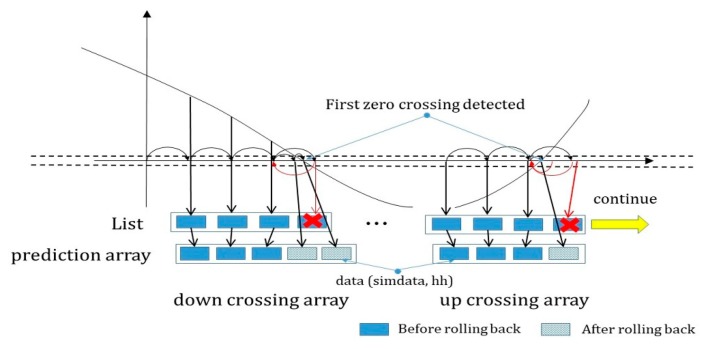
Zero crossing prediction with up and down crossing array.

In the case of ZC repeating with such a pattern, a “list” is created to store a certain number of simulation values continuously every time the simulation is performed in order to apply the step size by predicting the next ZC, as shown in Figure 10. During the first small repetition cycle $T_{p1}$, the simulation values before the occurrence of ZC are continuously stored in the “list”. Then, when down crossing or up crossing occurs, they are stored in the front part of the array space designed to predict each down/up crossing. The simulation values and step size *(h)* values from the start to the end of rollback are stored next in continuation. Because this study assumed the use of the bisection algorithm, the step size change while performing the rollback is always smaller than 1, i.e., $2^{-n}($n >= 1).

After ZC, the current simulation value is compared with the first value in the down/up crossing array saved previously to check if they are the same. If they have the same value, the remaining part of the array are all compared as well, and if all values are consistent, the step sizes saved in the previously performed rollbacks are applied sequentially, thereby preventing unnecessary rollbacks.

Secondly, in the case of ZC occurring repeatedly, the simulation graph has a continuous constant value in each section, but the value changes to a different value cyclically, as shown in Figure 9.

In the case of ZC repeating with such a pattern, the following processes (Algorithm 3) are performed to predict the next ZC. First, the current simulation value is compared with the previous value to check if they are the same. When the same value is maintained continuously, the current value is saved in the array storing the “level history” and the array index is increased. Here, the starting time of the level was saved, and afterwards, when the ZC reappeared and the simulation value changed, the ending time of the level was saved as well. Furthermore, the duration from the starting time to the ending time of the level was saved.
**Algorithm 3:** level zero crossing algorithm
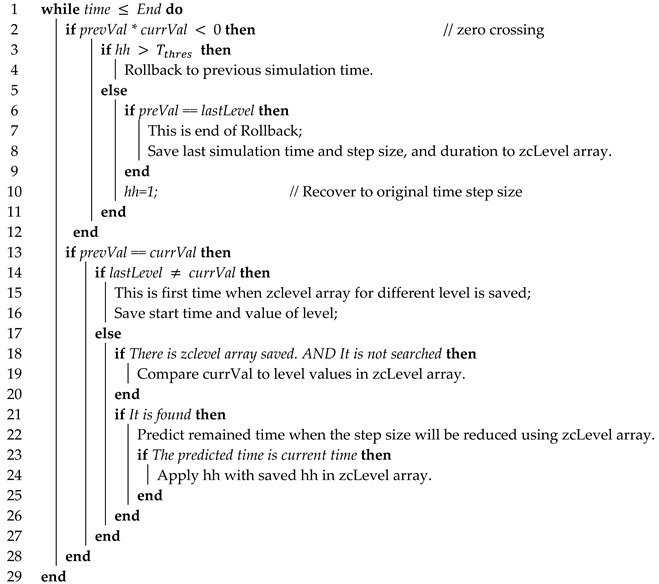


If the current simulation value is the same as the first value of the previous array, a search is performed to check whether the previously saved level value is in the array. If the level value exists, the saved duration time is added to the starting time of the current level in order to predict the time when ZC will occur in the future. If the remaining predicted ZC time is smaller than the current step size, the simulation is performed by changing the remaining time to the current step size, thereby moving to the timepoint immediately before the occurrence of ZC. Next, the previously saved step size value is applied to the next step size. If the step size is applied, the rollbacks can be reduced in the case of ZC occurrence. However, if ZC does not occur, the simulation is performed by reverting to the original step size.

## 4. Distributed CPS Model Based on Hybrid Modeling

### 4.1. EV Co-Simulation Model

**Figure 11:**
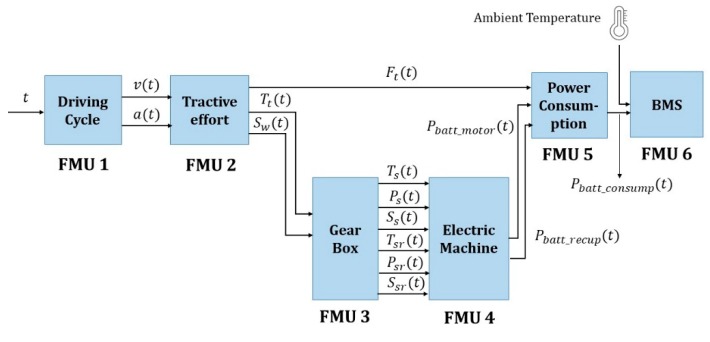
Functional mock-up interface-based electric vehicle co-simulation model.

The EV co-simulation model was used to simulate the measurement of the SOC of batteries according to the driving cycle. Figure 11 presents a block diagram of the co-simulation model, showing the input and output of each FMU. As shown in the figure, each subsystem implements an FMU of co-simulation type and performs the compiling using the FMU SDK tool and each FMU calculates and outputs a simulation value based on the input value. Every FMU was implemented using fmiSetFMUState and fmiGetFMUState to provide the rollback function, and the performance of the proposed MAs was tested through the co-simulation. In previous studies [36,37,38], the components of EV were modeled mathematically and based on these, simulations were performed to particularly measure battery consumption according to the driving cycle inputted in the EV model. Herein, the modeling of the subsystem through a mathematical model by the FMU is explained.

#### 4.1.1. Driving Cycle FMU

This FMU calculates the EV speed and acceleration value by time. The calculation formula is as follows. In other words, if the vehicle’s driving cycle is referenced, the speed value by current time *(s)* can be obtained. However, as this value is a discrete value of the second unit, a continuous simulation was facilitated through the following formula for each section by referencing the driving cycle. Here, if the acceleration *a(t)* is not provided in the driving cycle, it can be simply calculated as follows.

(1) $$v\left(t\right)=v_{0}+a\times t$$

(2) v(t)=v(k)+a(k)×t (k≤t<k+1)

(3) a(k)=v(k+1)−v(k) (k=0, 1,2,…(sec))a

#### 4.1.2. Tractive effort FMU

Next, the following is a formula used in the FMU model to find the vehicle’s tractive effort or force $F_{t}$, traction torque $T_{t}$ and traction power $P_{t}$. Based on these, the vehicle wheel’s angular velocity and revolution per minute (RPM) are determined.

(4) $$F_{t}=F_{rr}+F_{ad}+F_{hc}+F_{la}+F_{wa}$$

(5) $$F_{rr}=\mu_{rr}mg$$

(6) $$F_{ad}=\frac{1}{2}\rho AC_{d}v^{2}$$

(7) $$F_{hc}=mgsin\left(\alpha \right)$$

(8) $$F_{la}=ma$$

(9) $$F_{wa}=0.05\times F_{la}$$

(10) $$T_{t}=F_{t}\times r_{w}$$

(11) $$P_{t}=F_{t}\times v$$

(12) $$\omega_{w}=\frac{v}{r_{w}}$$

(13) $$S_{w}=\frac{30}{\pi }\;\times \;\omega_{w}$$

#### 4.1.3. Gear Box FMU

For a gear box model, the following formulas are used to calculate the electric machine (electric motors, electric generators)’s shaft power ($P_{s}$) and shaft torque ($T_{s}$) based on the vehicle’s tractive effort or force. If the tractive force is a negative number, i.e., If brake is applied, power is generated by regenerative braking; therefore, a negative value is obtained when calculated with the following formula:

(14) $$T_{s}=\;\frac{T_{t}}{\eta_{g}\times G}\;(P_{t}>0)$$

(15) $$T_{sr}=\;-\eta_{g}\times \;\frac{T_{t}}{G}\;(P_{t}<0)$$

(16) $$S_{s}=S_{sr}=G\times S_{w}$$

(17) $$P_{s}=T_{s}\times S_{s}\times \frac{\pi }{30}$$

(18) $$P_{sr}=T_{sr}\times \;S_{s}\times \;\frac{\pi }{30}$$

#### 4.1.4. Electric-Machine FMU

We used an induction machine (asynchronous machine) to model the electric machine and assumed that this model used an AC-DC converter for regenerative braking mode [41,42]. The efficiency map data determined by this model and the electric machine’s speed and torque data were used to obtain the efficiency of power consumed in the motoring mode and the efficiency of power generated in the recuperation mode, which are indicated by $\eta_{m}$ and $\eta_{r}$, respectively. Then, based on these, the power consumed in the motoring mode and the power generated in the recuperation mode were calculated by the following formulas. The Lookup2d function was implemented in the FMU. This function performs a role similar to that of lookup2d in MATLAB/Simulink. We calculated efficiency according to torque and the speed of the electric machine using efficiency curves given in [36].

(19) Efficiency(η)=lookup2d(Torque(Nm), Speed(rpm))

(20) $$\eta_{m}=\mathrm{lookup}2d\left(T_{s},\;S_{s}\right)$$

(21) $$\eta_{r}=\mathrm{lookup}2d\left(T_{sr},\;S_{sr}\right)$$

(22) $$P_{bm}=\frac{P_{s}}{\eta_{m}}$$

(23) $$P_{br}=\eta_{r}\times \;P_{sr}$$

#### 4.1.5. Power Consumption FMU

Suppose auxiliary load $P_{aux}$ is the power used for other purposes such as radio or heater, not the power used for tractive force through vehicle wheels in an EV. Then, this model is for a formula that calculates the total power consumed in the EV. If the tractive force is a positive number, then the total power consumed in the EV is the sum of the auxiliary power consumption and the power consumed in the motoring mode for wheel rotation. If the tractive force is a negative number, then it is the sum of the auxiliary power consumption and the power generated in the recuperation mode because the vehicle is in a braking situation.

(24) $$P_{bc}\left(t\right)=\{\begin{array}{c} P_{bm}\left(t\right)\;+\;P_{aux},\;\;\;\;F_{t}\left(t\right)>0\\ P_{br}\left(t\right)\;+\;P_{aux},\;\;\;\;F_{t}\left(t\right)<0 \end{array}$$

#### 4.1.6. Battery Management System (BMS) FMU

The battery management system model consists of a battery pack and a module that measures the SOC and SOH of the battery [43]. A battery current $I_{B}$ can be obtained through the battery’s no-load voltage $E_{B0}$, internal resistance $R_{B0}$, and power consumption calculated by (24). If this is integrated, the total consumed electric charge Q can be obtained. Furthermore, the battery cell capacity C may change depending on the ambient temperature, as revealed by the following formula [34]. Through this, the current SOC value of the battery can be finally be obtained.

(25) $$I_{B}\left(t\right)=\frac{E_{B0}}{2\times R_{Bi}}-\sqrt{{\left(\frac{E_{B0}}{2\times R_{Bi}}\right)}^{2}-\frac{P_{bc}}{R_{Bi}}}$$

(26) $$Q\left(t\right)=\int_{0}^{t}I_{B}dt$$

(27) $$C=C_{0}*\left(1+alphaC\times \left(T_{current}-T_{ref}\right)\right)$$

(28) $$SOC\left(t\right)=\frac{C-Q\left(t\right)}{C}$$

In addition, the SOH of the battery can be calculated as follows:

(29) $$SOH=1-SOS=SOH_{c}\times SOH_{z}$$

For a new battery, $SOH_{c}=1$ and $SOH_{z}=1$ therefore, also SOH=1. In this paper, it is assumed that the value of SOH is 1.

### 4.2. Energy Conversion Chain of EV Co-Simulation Model

**Figure 12:**
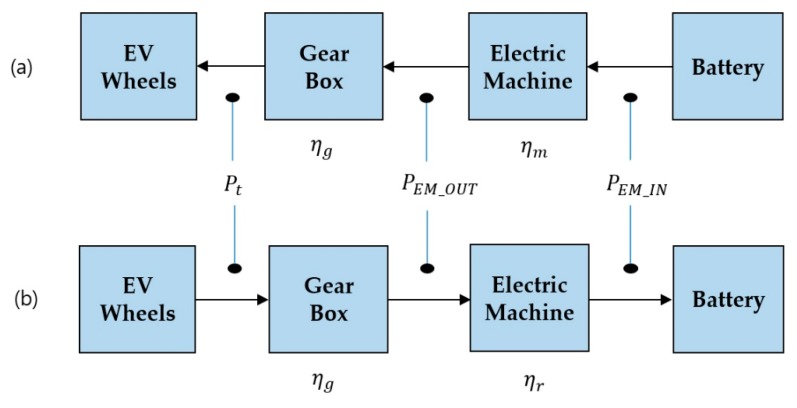
Energy conversion chain of each modes; (**a**) motoring mode, (**b**) regenerative braking mode.

The EV simulation model has different energy conversion chains because of different energy flows depending on the motoring and the regenerative mode [37]. In Figure 12, the energy required to move the EV for 1 s is the same as $P_{t}$. First, in the case of motoring mode, the energy of each part is calculated as follows:

(30) $$P_{EM\_IN}=\;\frac{P_{EM\_OUT}}{\eta_{m}}$$

(31) $$P_{EM\_OUT}=\;\frac{P_{t}}{\eta_{g}}(P_{t}>0)$$

Next, in the regenerative braking mode, the energy of each part is calculated as follows:

(32) $$P_{EM\_IN}=\;P_{EM\_OUT}\times \eta_{r}$$

(33) $$P_{EM\_OUT}=\;P_{t}\times \eta_{g}\;(P_{t}<0)$$

### 4.3. Distributed CPS Simulation Model Considering Sensor Information

**Figure 13:**
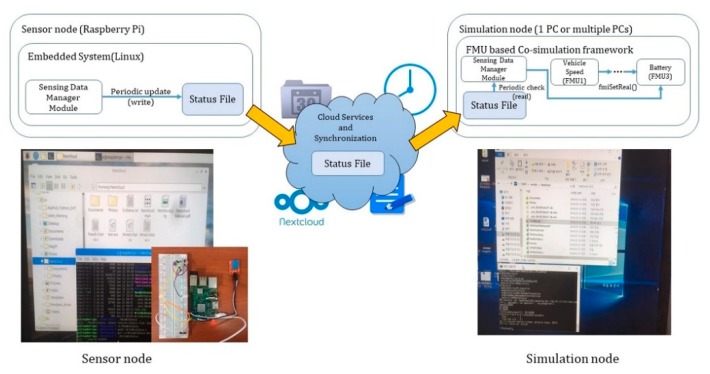
Simulation model including sensor information.

Moreover, the EV model can be simulated with the following structure in order to use real-time sensing data. In other words, the F-DCS simulates the EV model and the sensor node collects sensed temperature data and saves them in a file, as shown in Figure 13. The data values saved in the file were applied to the FMU in real-time through a cloud service in order to perform the simulation.

## 5. Simulation Experiments and Analysis

We experimentally validated the EV simulation model with the proposed algorithms to verify the results by employing driving cycle data. In the test, the new European driving cycle (NEDC), where $V_{max}$ equals 120 km/h and $V_{avg}$ equals 32 km/h, was adopted as a standard driving cycle. The NEDC, one of the standard driving cycles that includes specifications of urban, rural, and highway driving conditions, is composed of four consecutive ECE-15 urban driving cycles (UDC) and one extra-urban driving cycle (EUDC). Although the NEDC was originally designed to assess gasoline-driven vehicles, it is now used to evaluate and measure the power consumption and driving range of diesel-driven, hybrid and electric vehicles. The NEDC reflects the necessary conditions for evaluating the EV system as it provides average velocity, certain ratio of braking time, and acceleration similar to those in actual driving. Therefore, it provides cases that closely resemble real-world situations in EV model simulations. We used FMU SDK [44] and OpenDDS [45] for implementing F-DCS. For these experiments, we used six personal computers and an Ethernet switch. Table 1 shows the EV simulation parameter setting values.

Several studies involving conventional EV simulations first modeled the subsystems of an EV mathematically, after which they implemented and simulated the models using MATLAB/Simulink. This approach allowed the power system and driving distance of the EV to be predicted more accurately [36,37,41,42]. In our study, we implemented the subsystems of an EV based on the mathematical modeling method of existing studies. Therefore, the entire model shown in Figure 11 was implemented and simulated with MATLAB/Simulink first. Then, the results were compared with the simulation results that were derived by applying the method proposed in this paper, thereby evaluating the accuracy of the proposed simulation.

**Figure 14:**
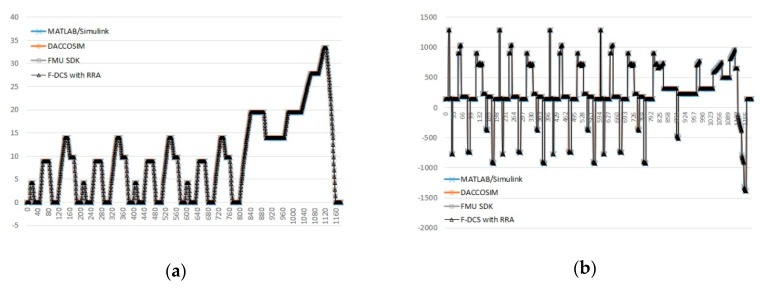

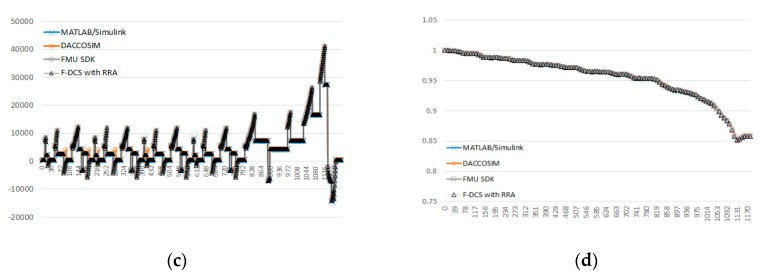
Results of EV simulation using driving cycle data; (**a**) Velocity (*m/s*) for time *t*, (**b**) tractive effort or force F_t_ (*N*), (**c**) power consumption from the battery P_bc_ (W), (**d**) SOC of the battery.

Figure 14 shows the results of EV model simulation for 1180 s considering the driving cycle. The results presented in Figure 14 appear to agree well with the results of a simulation based on MATLAB/Simulink [46]. This is because it adopts the driving cycle data where most of the average velocities and key values are the same.

Furthermore, to quantitatively measure the simulation accuracy of the proposed method, the mean absolute percentage error (MAPE) value of the simulation values was determined as follows:

(34) $$MAPE\left(\%\right)=\;\frac{1}{n}\;\overset{n}{\underset{i=1}{\sum }}\left|\frac{\hat{x_{i}}-x_{i}}{x_{i}}\right|\times 100$$

where n is the number of simulation data values, $\hat{x_{i}}$ is the *i*th simulation result when a certain algorithm was used, and $x_{i}$ is the *i*th simulation value when MATLAB/Simulink was used. For the simulation data, the values measured in 1 s increments from 0 to 1180 s were used to evaluate the performance of the proposed algorithm. Because the MAPE can be applied only when the value of $x_{i}$ is not 0, as shown in the above equation, we measured the simulation results *F*_t_, *P*_bc_, and *SOC* that did not have a value of 0 in the simulation section.

The simulation values of FMU SDK and F-DCS with RRA, which are provided in Table 2, are less than 5%. Furthermore, the two algorithms yielded the same MAPE values, and this is because the F-DCS with the RRA algorithm was implemented on the basis of FMU SDK and the repeating sections were removed while maintaining the same simulation accuracy of FMU SDK. The MAPE values of DACCOSIM were smaller than those of F-DCS, and it was shown that accurate simulation is feasible.

Next, each algorithm was evaluated by using the following equation to measure the zero crossing point error (ZCPE), which indicates the error in the simulation time for ZCP. Here, *m* is the frequency of ZCP that occurs in all simulation sections.

(35) $$Avg\;ZCPE\;=\;\frac{\sum_{i=1}^{m}T_{n+1}-T_{n}}{m}\;\left(for\;all\;ZCP\right)$$

The simulation results in Table 3 indicate that the average ZCPE of DACCOSIM and FMU SDK is the same as the step size. This is because in the case of a fixed step size, the time error for ZCP is proportional to the step size, as shown in Figure 9. However, in the case of F-DCS with RRA, it is confirmed that the simulation can be performed for ZCP with a value smaller than the time threshold value by using the bisection algorithm.

**Figure 15:**
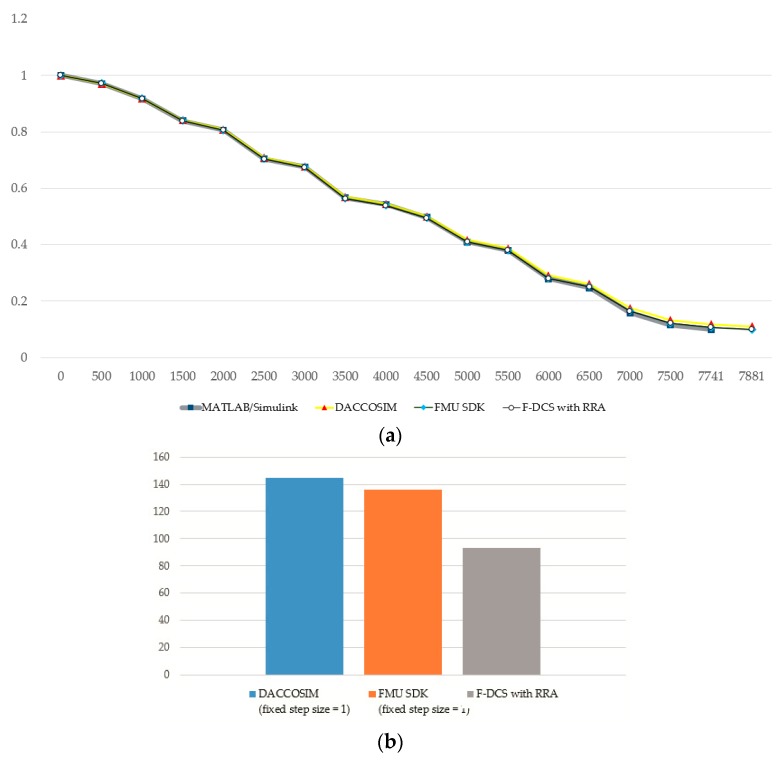
Results of model validation; (**a**) result of battery SOC, (**b**) average total simulation time (sec).

The simulation results using the NEDC driving cycle data, where the validation procedure with the measurement of the SOC value changing with simulation duration until the value of 0.1 was applied, is shown in Figure 15. In the validation with the application of the F-DCS with RRA for effectively reducing the overall simulation time, the calculated amount of battery SOC consumed was confirmed to agree with the results of general simulation cases (MATLAB/Simulink, DACOSSIM, FMU SDK default co-simulation algorithm [47]). An examination of the simulation result in Figure 15a reveals that the resulting battery SOC was almost the same as that of other conventional simulation tools. This result verifies the accuracy of the simulation measurement of the battery SOC of the proposed F-DCS. The simulation running time was evaluated in comparison with other FMI-based simulation tools. As shown in Figure 15b, the simulation time required by F-DCS with RRA was more than 30% less than that required by other methods. This means that if a driving cycle pattern with repetitive cycles exists in the simulation, it is safe to perform the simulation by proceeding with the rollback using that pattern. In addition, comparing the values of the stored rollback pattern information with the values generated during the progress of the simulation, looking for mapped values and applying them can improve efficiency in terms of simulation time.

**Figure 16:**
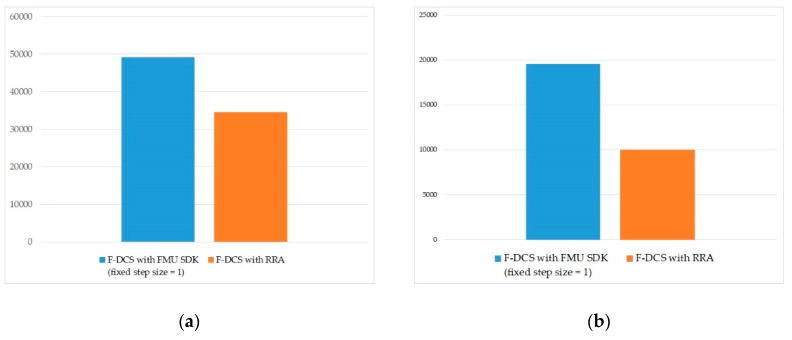
Results of data distribution service (DDS) validation; (**a**) DDS data message, (**b**) DDS control message.

Figure 16a shows the results of the performance of the number of DDS data message transmissions and in an environment that considers distributed co-simulation. As data are transmitted in the pub/sub format, as described by the distributed CPS simulation frameworks in Section 4, the number of messages generated by a simulation with a connected structure, as shown in Figure 11, can be calculated by the following formula.

(36) $$Total\;data\;message=Simulation\;loop\;num_{algorithm}\times \;num_{link}$$

(37) $$Total\;control\;message=Rollback\;num_{algorithm}\times (num_{node}-1)$$

Figure 16b shows that the result of DDS control messages decreased by more than 45%. With the FMU SDK algorithm, the occurrence of message generation increases significantly as all nodes that subscribe to a rollback receive a message containing the rollback information when the rollback occurs. By applying the F-DCS with RRA algorithm, the time for exchanging messages with the progress of simulation appears to be reduced by more than 30%, according to the reduction of the number of rollbacks using the patterns.

**Figure 17:**
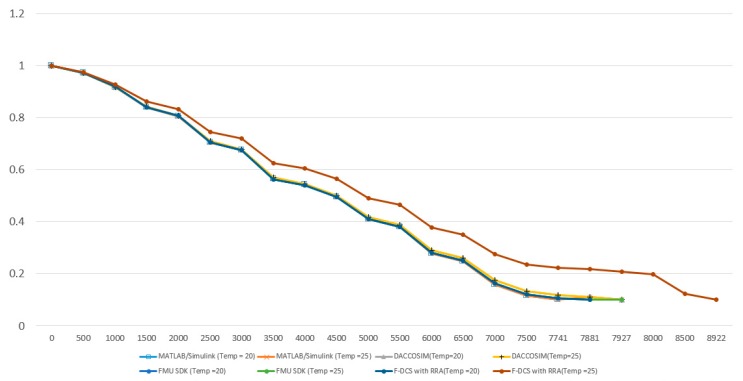
Test results on distributed co-simulation linked with sensor information.

Finally, the result of applying the sensor to the simulation and linking it is demonstrated in Figure 17. The simulation test was conducted using the test model shown in Figure 11 and a wide range of ambient temperatures was used for the information generated from the sensor. The simulation test was performed by using the measurement environments of 20 °C and 25 °C for the temperature measured with the sensor. In the results of battery SOC, the sensor information was not reflected without the F-DCS; nevertheless, the proposed algorithm could obtain simulation results by reflecting the sensor information value.

## 6. Conclusions

Co-simulation has significant potential in terms of improving the accuracy and efficiency of CPS simulations. However, because modeling and simulation differ between industrial areas, it is reasonable to apply appropriate techniques to each area. This paper presents a DDS middleware-based distributed co-simulation framework and proposes a method of determining the step size that can be commonly applied in all FMUs. When a repetitive pattern was observed in the frequently used driving cycle in EV simulation, simulations were performed by predicting the pattern. Based on this method, the proposed algorithms facilitated more efficient simulations while still maintaining conventional accuracy.

The proposed algorithms were applied to the EV simulation model and the results confirm that the proposed algorithms improved the performance by reducing the simulation time by over 30% compared to conventional algorithms. Furthermore, real-time data were received from the sensor node and applied to the EV simulation model. The results confirm that the real environment could be considered in the simulations. If an algorithm applicable to models of other areas in addition to the EV model in this study were developed, it would become the foundation of digital twin research.

## Figures and Tables

**Table 1:** EV simulation parameter setting values.

	Description	Value (Default)
*m*	Mass of the vehicle (kg)	1000
$r_{w}$	Wheel radius (m)	0.2736
*g*	Gravity acceleration (m/s^2^)	9.81
ρ	Air density	1.2
*A*	Front area of vehicle (m^2^)	2.36
α	Angle of driving surface (rad)	0
$\mu_{rr}$	Rolling resistance coefficient	0.015
*C*_d_	Aerodynamic drag coefficient	0.3
$\eta_{g}$	Gearbox efficiency	0.98
*G*	Gearbox ratio	8.59
*C*_0_	Initial capacity (C)	720000
*R*_bi_	Internal resistance (Ω)	0.008
*E*_b0_	Open-circuit voltage (V)	53.6
*alphaC*	Linear temperature coefficient of capacity (K^−1^)	0.03 (0)
*T_ref_*	Reference temperature	20
*SOH_c_*	Capacity state of health	1
*SOH_z_*	Impedance state of health	1

**Table 2:** MAPE (%) of simulation values.

Simulation Value	DACCOSIM	FMU SDK	F-DCS with RRA
*F*_t_	6.7	4.8	4.8
*P*_bc_	11.7	1.25	1.25
*SOC*	0.075	0.66	0.66

**Table 3:** Average zero crossing point error (ZCPE) of simulation values.

Simulation Value	DACCOSIM/FMU SDK			F-DCS with RRA
	Fixed Step Size			Adaptive Step Size (*T*_thres_ = 0.0001)
	1	0.01	0.0001	
	Average ZCPE			
*F*_t_	1	0.01	0.0001	0.000061
*P*_bc_	1	0.01	0.0001	0.000061

## Nomenclature

**Abbreviations**	
CPS	cyber physical system
EV	electric vehicle
ICT	information and communication technology
CPESs	cyber physical energy systems
WAN	wide area network
ZC	zero crossing
ZCP	zero crossing point
ZCPE	zero crossing point error
FMI	Functional mock-up interface
API	application programming interface
FMU	functional mock-up unit
IP	intellectual property
MA	master algorithm
DDS	data distribution service
OMG	object management group
IoT	internet of things
RPM	revolution per minute
BMS	battery management system
MAPE	mean absolute percentage error
NEDC	new European driving cycle
UDE	urban driving cycle
*SOC*	State of charge
*SOH*	State of health
*SOS*	State of sickness
**Variables**	
*v*	Velocity of the vehicle, m/s
*A*	Frontal area of the vehicle, m^2^
*a*	Acceleration of the vehicle, m/s^2^
*F*_t_	Traction force of the vehicle, N
*F*_rr_	Rolling resistance force of the wheels, N
*F*_ad_	Aero dynamic drag, N
*F*_hc_	Hill climbing force, N
*F*_la_	Force required to give linear acceleration, N
*F*_wa_	Force required to give angular acceleration to the rotating motor, N
$\mu_{rr}$	Coefficient of rolling resistance
*m*	Vehicle mass, kg
*g*	Gravity acceleration, m/s^2^
ρ	Density of the air
*C*_d_	Aerodynamic drag coefficient
α	Angle of the driving surface, rad
*r*_w_	Wheel radius, m
*P*_t_	Traction power, W
*w*_W_	Angular velocity of the wheels, rad/s
*S*_W_	Angular velocity of the wheels, rpm
*T*_t_	Traction torque, Nm
*T*_s_	Shaft torque of electric machine (motoring mode), Nm
*T*_sr_	Shaft torque of electric machine (regenerative braking mode), Nm
$\eta_{g}$	Gearbox efficiency g
G	Gear ratio of differential
*S*_s_	Shaft angular velocity of electric machine (motoring mode), rpm
*S*_sr_	Shaft angular velocity of electric machine (regenerative breaking mode), rpm
*P*_s_	Shaft power of electric machine (motoring mode), W
*P*_sr_	Shaft power of electric machine (regenerative breaking mode), W
$\eta_{m}$	Efficiency of power consumed (motoring mode)
$\eta_{r}$	Efficiency of power generated (regenerative breaking mode)
*P*_bm_	Power consumed by electric machine (motoring mode), W
*P*_br_	Power generated by electric machine (regenerative breaking mode), W
*P*_aux_	Power consumed by auxiliary loads, W
*P*_bc_	Total power consumed in the EV, W
*E*_b0_	Open-circuit voltage of the battery, V
*R*_Bi_	Internal Resistance of the battery, Ω
*Q*	Total charge of the battery
*I*_B_	Battery current,
*C*	Battery capacity, Ah
*alphaC*	Linear temperature coefficient of the capacity C, K^−1^
*T_ref_*	Reference temperature, K
